# Global transcriptome analysis reveals extensive gene remodeling, alternative splicing and differential transcription profiles in non-seed vascular plant *Selaginella moellendorffii*

**DOI:** 10.1186/s12864-016-3266-1

**Published:** 2017-01-25

**Authors:** Yan Zhu, Longxian Chen, Chengjun Zhang, Pei Hao, Xinyun Jing, Xuan Li

**Affiliations:** 1 Key Laboratory of Synthetic Biology, CAS Center for Excellence in Molecular Plant Sciences, Institute of Plant Physiology and Ecology, Shanghai Institutes for Biological Sciences, Chinese Academy of Sciences, Shanghai, 200032 China; 2 Germplasm Bank of Wild species in Southwest China, Kunming Institute of Botany, Chinese Academy of Science, Kunming, Yunnan 650201 China; 3 Key Laboratory of Molecular Virology and Immunology, Institute Pasteur of Shanghai, Chinese Academy of Sciences, Shanghai, 200031 China; 4 University of Chinese Academy of Sciences, Beijing, 100049 China

**Keywords:** *S. moellendorffii*, Lycophyte, Vascular plant, Transcriptome, Long noncoding RNA, Alternative splicing, Regulation, Transcription

## Abstract

**Background:**

*Selaginella moellendorffii*, a lycophyte, is a model plant to study the early evolution and development of vascular plants. As the first and only sequenced lycophyte to date, the genome of *S. moellendorffii* revealed many conserved genes and pathways, as well as specialized genes different from flowering plants. Despite the progress made, little is known about long noncoding RNAs (lncRNA) and the alternative splicing (AS) of coding genes in *S. moellendorffii*. Its coding gene models have not been fully validated with transcriptome data. Furthermore, it remains important to understand whether the regulatory mechanisms similar to flowering plants are used, and how they operate in a non-seed primitive vascular plant.

**Results:**

RNA-sequencing (RNA-seq) was performed for three *S. moellendorffii* tissues, root, stem, and leaf, by constructing strand-specific RNA-seq libraries from RNA purified using RiboMinus isolation protocol. A total of 176 million reads (44 Gbp) were obtained from three tissue types, and were mapped to *S. moellendorffii* genome. By comparing with 22,285 existing gene models of *S. moellendorffii*, we identified 7930 high-confidence novel coding genes (a 35.6% increase), and for the first time reported 4422 lncRNAs in a lycophyte. Further, we refined 2461 (11.0%) of existing gene models, and identified 11,030 AS events (for 5957 coding genes) revealed for the first time for lycophytes. Tissue-specific gene expression with functional implication was analyzed, and 1031, 554, and 269 coding genes, and 174, 39, and 17 lncRNAs were identified in root, stem, and leaf tissues, respectively. The expression of critical genes for vascular development stages, i.e. formation of provascular cells, xylem specification and differentiation, and phloem specification and differentiation, was compared in *S. moellendorffii* tissues, indicating a less complex regulatory mechanism in lycophytes than in flowering plants. The results were further strengthened by the evolutionary trend of seven transcription factor families related to vascular development, which was observed among four representative species of seed and non-seed vascular plants, and nonvascular land and aquatic plants.

**Conclusions:**

The deep RNA-seq study of *S. moellendorffii* discovered extensive new gene contents, including novel coding genes, lncRNAs, AS events, and refined gene models. Compared to flowering vascular plants, *S. moellendorffii* displayed a less complexity in both gene structure, alternative splicing, and regulatory elements of vascular development. The study offered important insight into the evolution of vascular plants, and the regulation mechanism of vascular development in a non-seed plant.

**Electronic supplementary material:**

The online version of this article (doi:10.1186/s12864-016-3266-1) contains supplementary material, which is available to authorized users.

## Background

*Selaginella moellendorffii*, a lycophyte, is a model plant to study the early evolution and development of vascular plants. The lycophytes diverged from the ancestor of vascular plants about 410 million years ago, and currently consist of clubmosses, quillworts and Selaginella [1, 2]. The lycophytes differ from the euphyllophytes, as they have not evolved flowers and seeds. For reproduction, the sporophytes release haploid spores that start an independent gametophyte generation. The lycophytes occupy a key phylogenetic position in the evolution of vascular plants. As the first and only sequenced lycophyte to date, the genome of *S. moellendorffii* was recently reported [3] to have a size of 212.6 Mbp, containing 22,285 coding genes. The *S. moellendorffii* genome revealed many conserved genes and pathways, as well as specialized gene sets, differing from flowering plants, for generation of secondary metabolites. Comparative genome analysis found substantial gain in new genes for the transition from a non-seed vascular to a flowering plant [3]. Despite the progress made in the studies of *S. moellendorffii* genome, the existing models of 22,285 coding genes have not been fully examined and validated with transcriptome data. Neither information about alternative splicing (AS) of coding genes, nor about long noncoding RNAs (lncRNA) is available.

Long noncoding RNAs (lncRNA) are transcribed in plants and animals with structures similar to those of mRNAs. Important functions of lncRNAs emerged as a critical player in regulation in a range of biological processes in animals [4, 5], but also were implicated in plant development and reproduction [6, 7]. Examples of lncRNA in plants include IPS1 and COLDAIR, which function to modulate miRNA (miR-399), or recruit protein PRC2 to silence FLC gene [8, 9]. Using gene chip and RNA-Seq technologies, lncRNAs were screened and investigated in angiosperms, e.g. *Arabidopsis* [10, 11], maize [12, 13], rice [14, 15] and wheat [16]. However, with little was known about lncRNA in lycophytes, the study was designed to uncover and characterized lncRNAs in *S. moellendorffii*.

The appearance of vascular tissues is a landmark event in the evolution of plants from aquatic to terrestrial. Vascular system consists of two major components, xylem and phloem tissues, which helps plants free themselves from the dependency of the aquatic environment by transporting and redistributing water and nutrients [17]. Meanwhile, they provide mechanical support to expand leaves to capture sunshine for photosynthesis. Development of the vascular system involves a multi-step process, including differentiation of procambium, elongation of tracheary elements and sieve cells, and secondary wall formation [18], which are regulated by some complex regulatory mechanisms [19]. Using *Arabidopsis thaliana* as a model, many important gene regulators were implicated in the initiation, development and regulation of the vascular system [20–23]. However, it became important for us to understand whether similar regulatory mechanisms and process are used, and how they operate in a non-seed primitive vascular plant, like *S. moellendorffii*. By analyzing the gene expression profiles and regulation in *S. moellendorffii* tissues, we hope to address these critical questions. And furthermore, by extending the comparison to those of non-vascular plants, chlorophyta and bryophyta, and angiosperms, we can gain insight into the evolution of regulatory elements and molecular mechanisms of the vascular system.

In the current study, a multi-tissue transcriptome analysis was designed to investigate the full gene contents in *S. moellendorffii* and characterize their expression profiles in differentiated tissue types, i.e. root, stem and leaf tissues. Our study was focused in five main areas: 1) the gene models of existing and novel coding genes; 2) the alternative splicing of coding genes; 3) long noncoding genes; 4) differential gene expression in tissues types; and 5) expression of genes related to vascular development in tissues types. Technically we applied the RiboMinus protocol in RNA isolation to maximize RNA species from *S. moellendorffii*, and created the strand-specific RNA-seq libraries in order to reconstruct directional RNA transcripts. We extensively refined the existing gene models, discovered novel coding genes and noncoding RNA (ncRNA), and characterized their alternative splicing (AS). We revealed the tissue-specific gene regulation in *S. moellendorffii*, and the expression profile of many important genes related to vascular development was further assessed and discussed.

## Results and discussion

### Collection and RNA sequencing of *S. moellendorffii* tissue samples

**Fig. 1:**
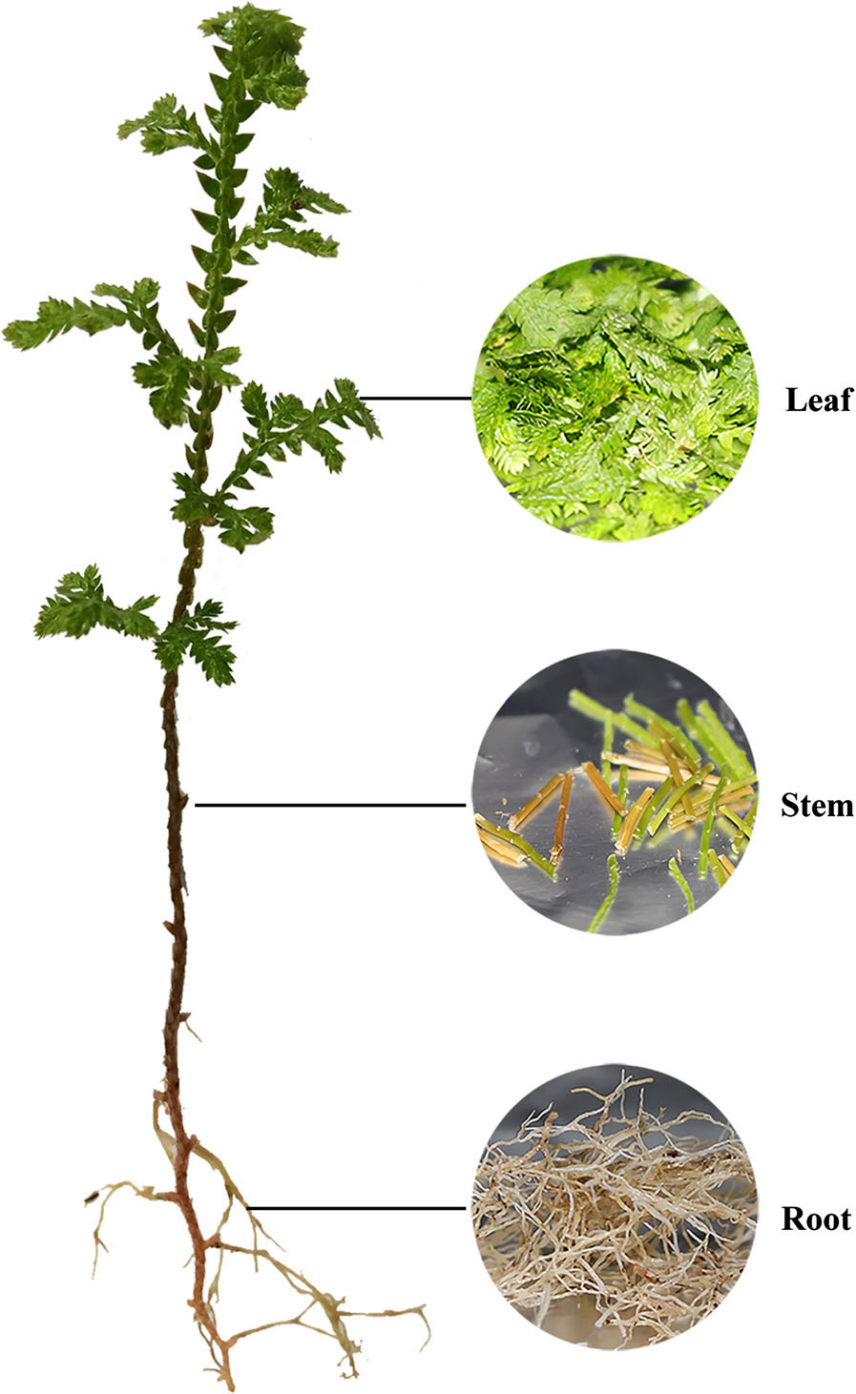
*S. moellendorffii* plant and three tissues collected in this study

To discover the full gene contents in *S. moellendorffii* and characterize their expression profiles, the study was designed to perform deep sequencing on RNA of differentiated tissues from *S. moellendorffii*. Root, stem and leaf samples of *S. moellendorffii* were first collected (Fig. 1), from which total RNA was isolated as described in Methods. Because regularly used PolyA+ RNA often had reduced RNA species, in order to have a more broad representation of RNA transcripts, the RiboMinus protocol [24] was used to remove rRNAs from total RNA. Then, to distinguish the strand from which RNA was transcribed, a strand-specific protocol [25] was employed to construct RNA-seq library for each tissue type. The RNA-seq libraries were sequenced using Illumina HiSeq2500 platform, with a paired-end read length of 125 base pairs (bp). A total of 176 million reads (approximately 44 Gbp) were obtained for all three *S. moellendorffii* tissues (Table 1). To determine the efficiency of the RiboMinus protocol, sampled reads were checked for rRNA sequence, which was found to represent ~0.03% of raw sequence reads. And, these reads were removed from our data. Then low-quality reads, adaptor or ambiguous sequences were filtered, retaining 161 million (39 Gbp; 91% of raw data) high-quality clean reads for subsequent analyses. The avail of the tissue specific RNA-seq data from *S. moellendorffii* provided us with an unprecedented opportunity to characterize the gene contents and their expression profile in a non-seed vascular plant.

**Table 1:** Overview of the sequencing and gene models of *S. moellendorffii*

	Total No.	Root	Leaf	Stem
Raw reads	176,439,664	53,984,797	59,076,216	63,378,651
Clean reads	161,588,604	47,787,568	52,573,643	61,227,393
Mapped reads	105,111,858	25,288,623	37,761,820	42,061,415
Existing gene models	22,285	15,038	15,433	15,133
Novel transcripts	20,882	19,454	19,900	19,675
Novel genes	7930	7435	7552	7524
Antisense TUs	1665	1552	1599	1567
lincRNAs	3660	3, 605	3609	3602
Antisense-lncRNA	762	720	746	723
Total coding genes	30,215	22,473	22,985	22,657

### Mapping RNA-seq reads and annotation of *S. moellendorffii* genome

To analyze the transcriptome profile of *S. moellendorffii* and its gene models, we first filtered reads from three tissues and mapped them separately to the *S. moellendorffii* reference genome. About 105,111,858 (~65%) filtered reads (paired-end and directional) were mapped, covering 21,569 (96.8%) of the 22,285 annotated genes in *S. moellendorffii* genome. 20,784 novel transcripts (Table 1) were identified, using Cufflinks (version 2.2.1) [26], based on the mapping results and existing gene models of *S. moellendorffii*. Among them, 12,841 transcripts were predicted to have coding potential with the CPC tool [27]. Using more stringent criteria having: 1) open reading frames >100 amino acids; and 2) homologous proteins/domains found in other organisms, 7930 transcripts (Additional file 1) of them were designated as high-confidence novel coding genes. For the rest, 121 transcripts were found to be rRNA, tRNA or precursors of microRNA based on Rfam [28] and miRBase [29] databases. The remaining 7822 transcripts (Additional file 2) represented noncoding RNAs (ncRNAs) newly discovered in *S. moellendorffii*. We selected 18 novel transcripts for RT-PCR validation, and 15 were found to produce PCR product of corrected size (Additional file 3).

The discovery of the high-confidence novel coding genes (7930) brings the total number of coding genes in *S. moellendorffii* to 30,215, a 35.6% increase over previously annotated genes for *S. moellendorffii*. Consequently, it increased the gene density to ~284 gene/Mb in *S. moellendorffii*, closer to that in *Arabidopsis* (310 gene/Mb) [30]. The coding gene transcripts have an average length of 1.5 Kb with 6 exons on average, compared to an average length of 0.7 Kb with 2.4 exons for lncRNAs in *S. moellendorffii* (detail below).

Among the 22,285 existing gene models of *S. moellendorffii*, 2461 (11.0%) were refined (Additional file 4), based on the supporting transcriptome data we obtained. Most of these refinement involved either new exons (1739, 71%) or changing boundary (722, 29%) for existing exons.

**Fig. 2:**
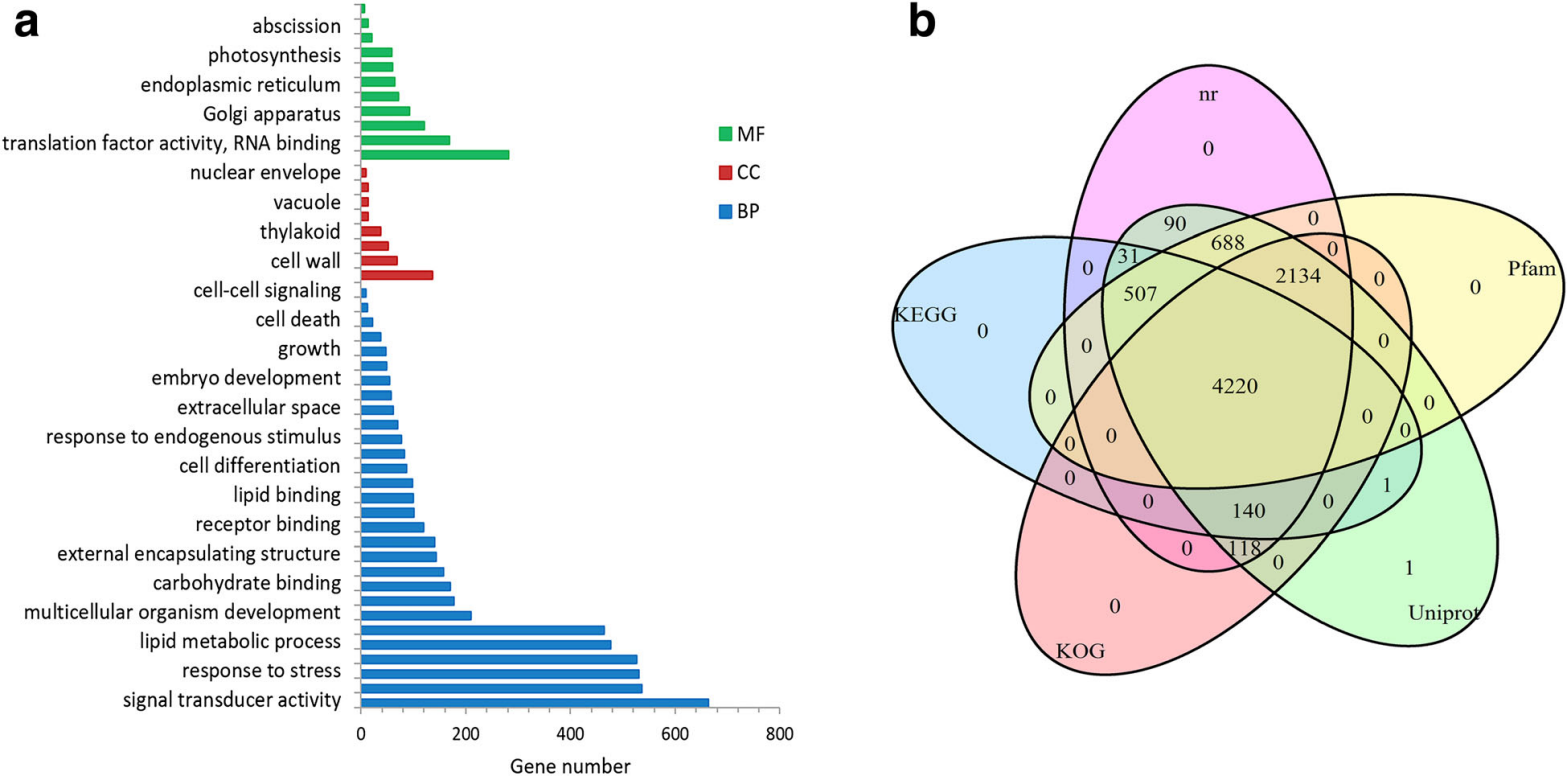
Function annotaions of novel coding genes in *S. moellendorffii*. **a** Gene Ontology (GO) functional classifications of novel coding genes. *MF* Molecular Function, *CC* Cellular Component, *BP* Biological Process. **b** Public database annotaions of novel coding genes. Each databse labeled with a colored oval

The CDS of the 7930 novel genes was annotated using Pfam, KEGG, Swiss-prot, KOG, and nr (Methods), and 2699 were found to have homologues in *Arabidopsis* (Additional file 1). The Gene Ontology (GO) analysis showed the novel coding genes occupied almost all the major functions of plant growth, development, metabolism, and stress response (Fig. 2a). While 4899 were linked to 10,782 KO terms involved in 346 KEGG pathways, 6612 have KOG annotation. Overall, 7928 novel coding genes can be defined by at least two of the five annotation methods (Fig. 2b). Additionally, five pathways (Primary bile acid biosynthesis, Indole alkaloid biosynthesis, Glucosinolate biosynthesis, Steroid degradation and beta-Lactam resistance) were first found in *S. moellendorffii*, according to the annotations of novel genes.

The ncRNAs (7822) (Additional file 2) were reported for the first time in *S. moellendorffii*. Among them, 6760 and 1062 were found in intergenic or antisense regions, respectively. However, with the help of strand-specific sequencing technology, 1665 transcripts (Additional file 5) were found to arise from the antisense strand of coding genes in *S. moellendorffii*, named as natural antisense transcript (NAT). The number of NATs was small compared to the 37,238 NATs previously reported in *Arabidopsis*, which accounted for 70% of all transcripts [31].

### Alternative splicing events in *S. moellendorffii*

Alternative splicing (AS) is a common mechanism to generate transcript isoforms in eukaryotes, greatly increasing proteomic diversity with limited gene number. No alternative splicing events were reported previously for *S. moellendorffii* genes. In the current study, we used TopHat (version 2.1.0) [32] to detect splice junction sites between exons of *S. moellendorffii* genes. Using mapped exon–exon junction reads based on gene models constructed/updated with Cufflinks tool, we identified 11,030 alternative splicing events for 5957 coding genes (Additional file 6), accounting for 19.7% of 30,215 total coding genes. The ratio is relatively low, compared to around 61% of total coding genes in *Arabidopsis* and 33% in rice [33, 34]. Similar phenomena were observed in lower vertebrates, in which fewer percentage of coding genes had AS events than those in higher vertebrates, e.g. mammals [35]. The tissue specificity of alternative splicing events in *S. moellendorffii* were further analyzed, with 450, 397 and 350 isoforms expressing specifically in root, stem and leaf, respectively (Additional file 6).

**Fig. 3:**
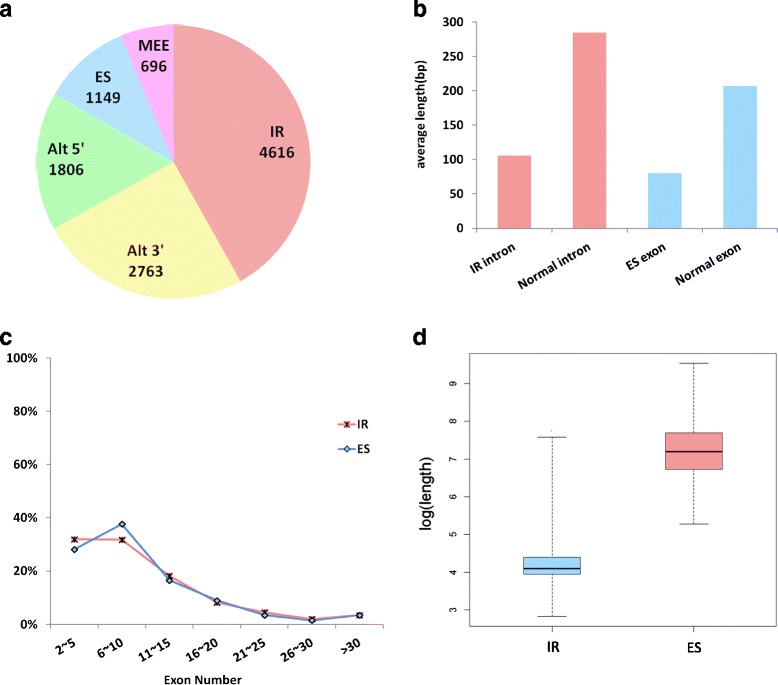
Properties of alternative splicing (AS) events in *S. moellendorffii* genome. *IR* intron retention, *ES* exon skipping, *Alt 5′* alternative 5′ splice site, *Alt 3′* alternative 3′ splice site, *MEE* mutually exclusive exons. **a** The numbers for each type of AS events. **b** Average length of exons from different sources. *Red bars* the average intron length of transcripts in IR events and normal transcripts. *Blue bars* the average exon length of transcripts in ES events and normal transcripts. **c** The percentage of transcripts with different exon numbers. *Red line* transcripts with IR events. *Blue line* transcripts with ES events. **d** The mRNA length of transcripts with IR and ES events

AS events can be classified into five types: intron retention (IR), exon skipping (ES), alternative 5′ splice site (Alt5′), alternative 3′ splice site (Alt3′), and mutually exclusive exons (MEE). In *S. moellendorffii*, 4616 IR events were identified, accounting for about 42% of all AS events (Fig. 3a). It proved what others had shown previously that IR events was the predominant type of AS events in plants [36]. Note that in *Arabidopsis* and rice, IR events accounted for about 40 and 47% of all AS events, respectively [33, 34]. The average length of spliced introns from IR events was estimated to be 106 bp, about one third of average length of all introns (285 bp) from *S. moellendorffii* (Fig. 3b). Similarly, it was also found that in rice, the average intron length from IR events was 183 bp, much smaller compared to the average intron size of 470 bp in general [34].

ES events varied in plants from 3% in *Arabidopsis* to 25% in rice [33]. The number of ES events in *S. moellendorffii* fell in the middle, accounting for 11% of all AS events (Fig. 3a). Most of the ES isoforms (88.9%) skipped one exon, whereas those skipping multiple exons were rare in *S. moellendorffii*. We observed 59 ES events skipping two exons, and 24 events skipping three exons. The average length of skipped exons was ~80 bp. In contrast, the average length of regular exons was 207 bp (Fig. 3b). We compared the exon number for transcripts with either IR or ES events, and found they had little difference (Fig. 3c).

However, transcripts with either IR or ES events had an apparent difference in transcript length in *S. moellendorffii* (Fig. 3d), with IR transcripts having much shorter total length than ES transcripts. The pattern for short and long transcripts to use IR or ES to form isoforms is clear but its reason remains puzzling.

Alt5′ and Alt3′ events in *S. moellendorffii* have similar frequencies of 16 and 25%, respectively (Fig. 3a). Though higher than those of *S. moellendorffii*, *P. patens* [37] had frequencies comparable between Alt5′ (21%) and Alt3′ (26%) events. Similarly, Alt3′ events (~15%) in *Arabidopsis* were more frequent than Alt5′ events (~7%). MEE events were the least frequent in *S. moellendorffii*, occupying ~6% of all AS events. Note the five types of AS events were also observed in lncRNA as discussed next.

### Long noncoding RNA in *S. moellendorffii*

The 7822 ncRNAs in *S. moellendorffii* (Additional file 2) were further analyzed. A set of long noncoding RNA (lncRNA) were identified using more strengthened criteria (Methods). As a result, 4422 lncRNAs were obtained from *S. moellendorffii*, and were classified into two groups based on their genomic location relative to coding genes: lncRNA in intergenic regions (lincRNA), and lncRNA on the anti-sense strand of coding genes (anti-lncRNA). There were at least 3660 lincRNA and 762 anti-lncRNAs in the *S. moellendorffii* genome. No lncRNAs located in intronic regions (intronic lncRNA) were found in *S. moellendorffii*.

**Fig. 4:**
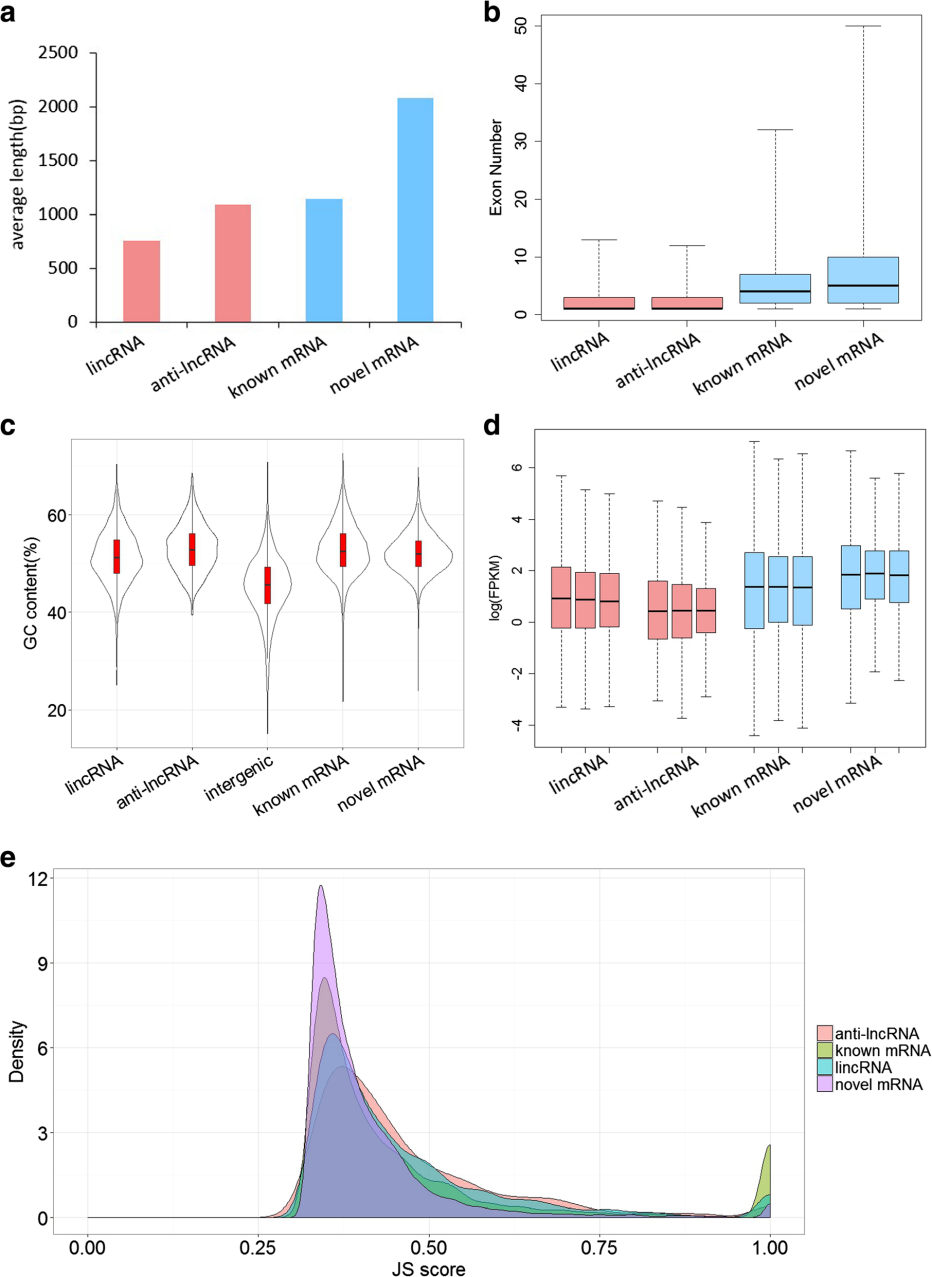
Properties of lncRNAs from *S. moellendorffii*. lincRNA: lncRNA located in intergenic region; anti-lncRNA: lncRNA located in antisense strand and overlapped to exons of mRNAs; intergenic: random intergenic sequence; known mRNA: mRNAs obtained from JGI database; novel mRNA: mRNAs of novel coding gene. **a** Average length of transcripts for lincRNA, anti-lncRNA, known mRNA and novel mRNA. **b** The number of exons per transcript. **c** GC content of lncRNAs and other types of transcripts, and the area size reflected the count of transcripts. **d** FPKM of transcripts in root, stem and leaf. **e** Tissue-specific expression of lincRNA, anti-lncRNA, known mRNA and novel mRNA, the X axis is the JS score, Y axis is the density

Both types of lncRNAs, lincRNAs and anti-lncRNAs, were shorter than mRNA in *S. moellendorffii* when compared to coding gene transcripts (Fig. 4a). The larger length of mRNAs was due to the higher number of exons that coding genes had in general. Existing coding gene transcripts (mRNA) in *S. moellendorffii* on average had 5.51 exons per transcript. The 7930 novel coding genes we identified in the current study on average had an even higher number of exons, 7.10 per transcript, reflecting the fact that longer transcripts were likely to be missed in earlier models without supporting RNA evidence. In contrast, lincRNA and anti-lncRNA on average had 2.47 and 2.16 exons per transcript, respectively (Fig. 4b). About 9.81% of lincRNAs and 9.82% of anti-lncRNAs were found to have alternative splicing (AS) in *S. moellendorffii*, compared to 19.7% of coding genes having AS. High GC content was usually associated with gene coding sequences [38]. In the current study, lincRNAs in *S. moellendorffii* were found to have a lower GC content (50%) compared to mRNAs (53%), but significantly higher than random intergenic regions (Fig. 4c). On the other hand, the GC content (53%) of anti-lncRNAs was similar to that of mRNAs (Fig. 4c), resulted from being reverse complement of mRNA.

The expression profile of lncRNAs was assessed by calculating their FPKM value (Fragment Per Kilobase per Million mapped reads) [26] in each tissue. Consistent with previous observations that lncRNAs were expressed at levels lower than mRNAs [39, 40], the expression ranges of both lincRNAs and anti-lncRNAs were lower than those of mRNAs in all three *S. moellendorffii* tissues (Fig. 4d). We further analyzed the tissue-specific expression of lincRNAs, anti-lncRNAs, and existing and novel mRNAs basing on the Jensen-Shannon (JS) score [41]. Unexpectedly, lincRNAs and anti-lncRNAs exhibited a degree of tissue-specific expression similar to that of mRNAs in root, stem, and leaf (Fig. 4e). They formed a clear contrast to lncRNAs in *Arabidopsis* and rice that had significantly different JS score from that of mRNAs [15, 39]. It is likely that lncRNAs in *Arabidopsis* and rice were differentially expressed across a greater number of specialized tissues, thus leading to increased JS score not observed in *S. moellendorffii* in the current study.

### Tissue-specific gene expression among *S. moellendorffii* tissues

**Fig. 5:**
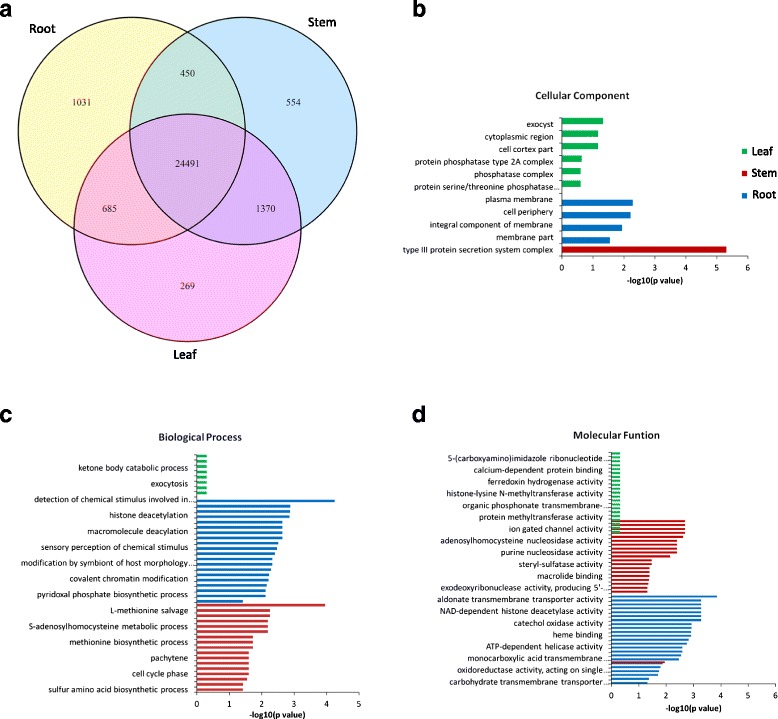
Genes expressed in different tissues and GO enrichment of tissue-specific genes. **a** Co-expressed and uniquely expressed genes in root, stem or leaf. **b**, **c**, **d** GO enrichment of tissue-specific genes in root, stem and leaf

*S. moellendorffii* represents an ancient linage of vascular plants, which evolved primary vascular tissues over 400 million year ago. To investigate the gene contents and expression pattern associated with the development of vascular tissues, we compared and characterized the tissue-specifically expressed coding genes among the *S. moellendorffii* tissues. The expression of coding genes was analyzed in root, stem and leaf. 26,656, 26,865 and 26,814 genes had expression level greater than 0.1 FPKM in root, stem and leaf tissues, respectively (Fig. 5a). The expression of roughly 24,491 genes, (81% of total coding genes) was shared by the three tissues in *S. moellendorffii*. On the other hand, there were 1031, 554, and 269 tissue-specific genes expressed in root, stem and leaf tissues, respectively (Fig. 5a, Additional file 7).

The GO enrichment analysis was performed on each tissue for tissue-specific genes (Fig. 5b, c, and d). Biological functions and processes, e.g. response to stress, response to stimulus, transportors, G-protein receptor signaling, histone deacetylase activity, etc. were enriched for root. The enriched functions and process for stem included detection of chemical stimulus, cellular metabolic compound salvage, amino acid biosynthesis, potassium channel activity, calcium activated cation channel activity, etc., whereas for leaf the enriched functions and processes were detection of chemical stimulus, glycoprotein biosynthesis, histone methylation, phosphotransferase activity, calcium-activated potassium channel activity, ion gated channel activity, etc.

The differential expression of lncRNA in the *S. moellendorffii* tissues was an intriguing subject. The differential expression analysis of lncRNA was similarly performed. 4135, 4119 and 4276 lncRNAs were expressed in root, stem and leaf tissues, respectively, whereas 3584 lncRNAs were shared by the three tissues. 194, 60, and 70 lncRNAs were specifically expressed in root, stem, and leaf tissues, respectively (Additional file 8), which may have important roles in regulation for tissue functions or development.

### Expression of critical genes for vascular development in *S. moellendorffii*

**Fig. 6:**
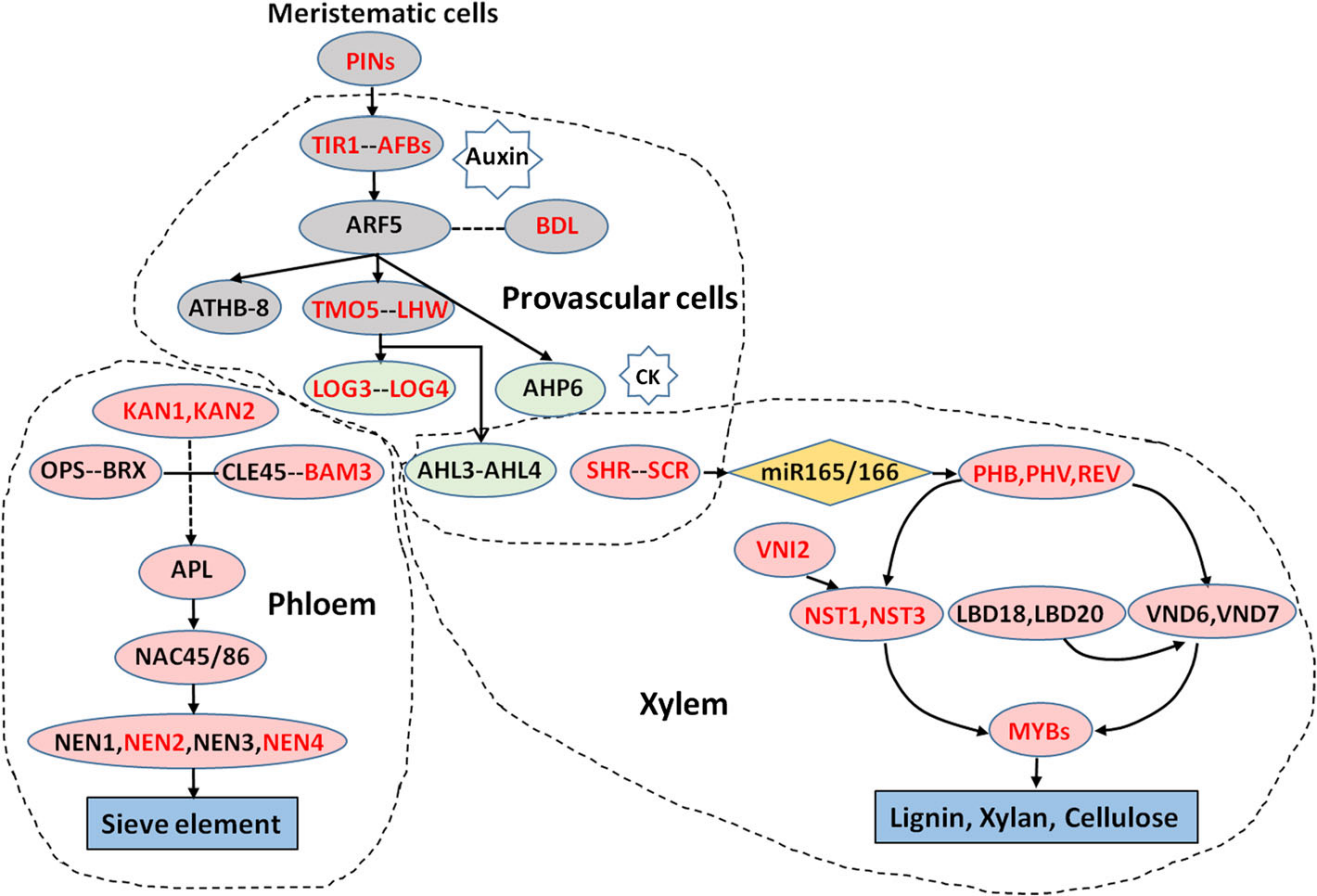
Important genes in vascular development pathways in *Arabidopsis* and *S. moellendorffii*. The three areas surrounded by *imaginary lines* are the stages of vascular development: *formation of provascular cells, xylem specification and differentiation, phloem specification and differentiation.* The genes of *Arabidopsis* are in the *ovals*, while the *red* ones are genes found in *S. moellendorffii.* The *black lines* with *arrows* represent targets or regulated relationships, while the *imaginary* represent the uncertain relationships. In the stage of provascular cells formation, the *ovals* filled in *grey* are genes related to auxin signals, while the *ovals* filled in *light green* are genes related to cytokinin signals. In the stage of xylem/phloem specification, the microRNA is labeled with *yellow rhombus*, and the compound or component of xylem/phloem are with *blue rectangle* background

The lycophytes are primitive vascular plant, and occupy a key phylogenetic position in the evolution of green plants. The key components of signaling and transcriptional regulation in vascular development in flowering plants have been investigated and revealed in model plants like *Arabidopsis* [19, 42, 43]. By comparing those from *S. moellendorffii* with the related *Arabidopsis* genes and pathways, we hoped to determine whether similar regulatory mechanisms and processes were also used in the lycophytes at the early stage of vascular plants, and if yes, how they operated in the primitive vascular plant. For the main stages of vascular development (Fig. 6), namely formation of provascular cells, xylem specification and differentiation, and phloem specification and differentiation, the involved genes from *S. moellendorffii* were mapped. The expression details for some of the critical genes were investigated and discussed.

#### The formation of provascular cells

**Fig. 7:**
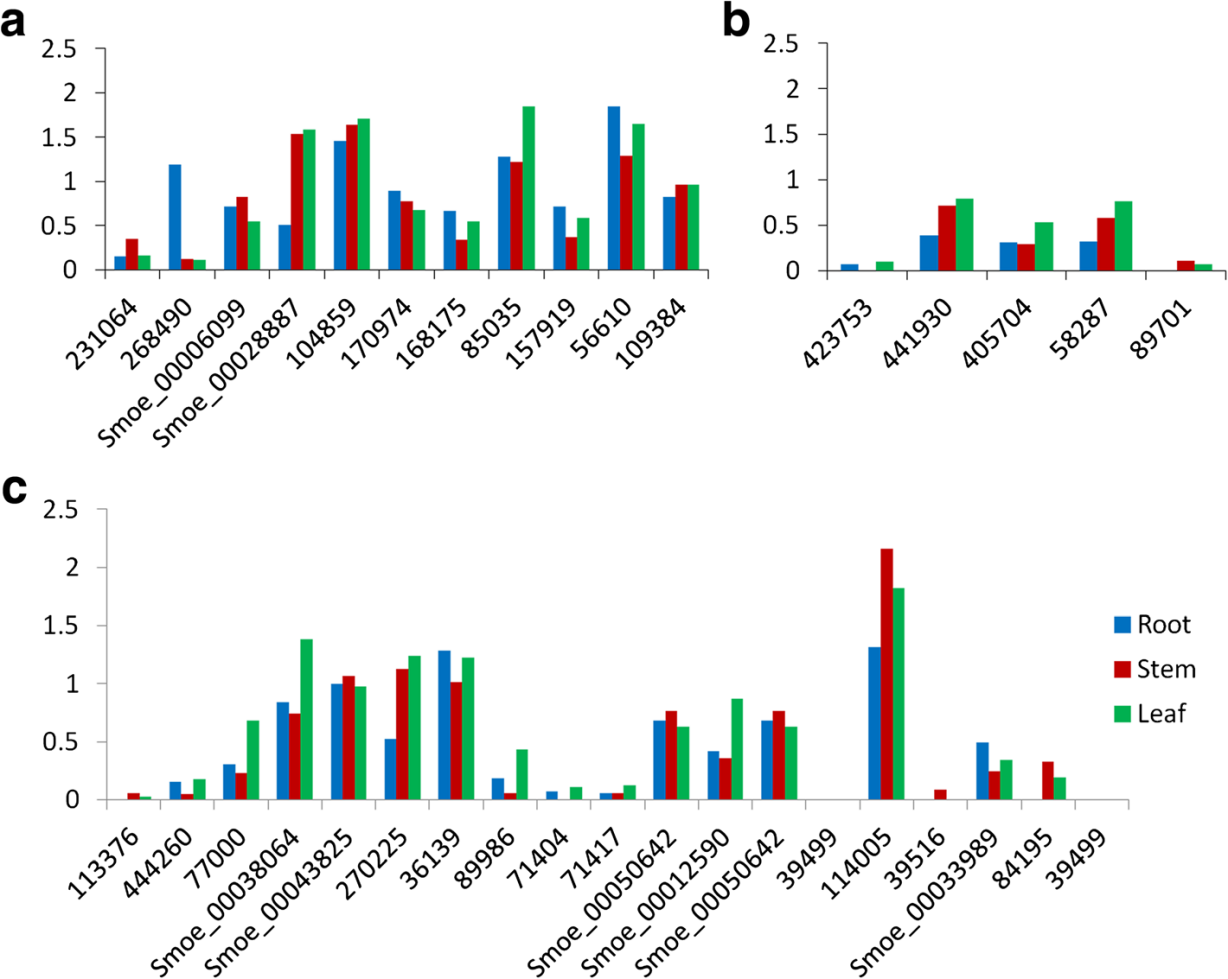
Expression levels of vascular development genes from root, stem and leaf in *S. moellendorffii*. **a**) Genes involved in “formation of provascular cells”; **b**) Genes involved in “xylem specification and differentiation”; **c**) Genes involved in “phloem specification and differentiation”. The expression level was calculated by log_10_ (FPKM + 1)

Vascular tissues in stem, leaf and other aboveground organs were originated from the shoot apical meristem. The phytohormones, i.e. auxin and cytokinin, signaled to initiate the formation of provascular cells. Members of PIN family were critical factors in auxin signaling, acting as auxin transporters in meristem and making the provascular cells sensitive and respond to auxin [44]. In *S. moellendorffii*, four homologues of PIN gene family, *231064*, *268490*, Smoe_00006099, and Smoe_00028887, were found to be expressed in root, stem and leaf (Fig. 7a; Additional file 9). Two of them, Smoe_00006099, and Smoe_00028887 were novel genes identified in the current study. Among the four, the PIN3 homologue (*268490*) expression was biased toward root, whereas PIN7 homologue (Smoe_00028887) was biased toward stem and leaf.

In *S. moellendorffii* tissues, TIR1 homologues (*104859*), AFB family members (*170974*, *168175*), and BDL homologue (*85035*) were found to be expressed in all three tissues (Fig. 7a; Additional file 9). ARF5 and BDL encode two important transcription factors, IAA24 and IAA12, controlling vascular formation during embryo development stage. However, ARF5 gene was not found in *S. moellendorffii*. Surprisingly, expression of LHW and TMO5, which are downstream targets of ARF5, were detected in all three tissues (*157919*, *56610*) (Fig. 7a; Additional file 9). How LHW and TMO5 transpond signals through the cascade in meristematic cells remains an intriguing and open question. Although homologues of both LOG4 and LOG3, the cytokinin signal response factors, existed in *S. moellendorffii*, only expression of LOG4 homologue was detected (Additional file 9). In the process of formation of provascular cells in *S. moellendorffii*, the missing factors may suggest a less complex regulatory mechanism than in flowering plants, e.g. *Arabidopsis*.

#### Xylem specification and differentiation

In *S. moellendorffii*, homologues of SHR and SCR, *113376* and *444260*, were expressed at low levels in root, stem and leaf (Fig. 7b; Additional file 9). While the homologues of four HD-ZIPIII family genes, PHB, PHV, REV, and ATHB-15, were found in *S. moellendorffii*, those of PHV and REV were novel genes (Smoe_00038064, Smoe_00043825) identified in the current study, expressing at moderate levels (Fig. 7b; Additional file 9). NAC transcription factor family plays key roles in the differentiation of xylem. A total of 7 NAC family members existed in *S. moellendorffii*, VNI2 (NAC083), VND1 (NAC037), VND2 (NAC076), VND4 (NAC007), NST1 (NAC043), NST2 (NAC066), and NST3 (SND1/NAC012). However, three of them, Smoe_00050642, Smoe_00012590, Smoe_00050642, were novel coding genes identified in current study. MYB transcription factor family can activate the biosynthesis of lignin, and their expressions were regulated by NAC family. In *S. moellendorffii*, 6 MYB family members, MYB20, MYB43, MYB46, MYB54, MYB61, and MYB85, were expressed in different tissues (Fig. 7b; Additional file 9). Particularly, MYB43 (*114005*) was expressed at high level in all three tissues, especially stem (Fig. 7b; Additional file 9). In *S. moellendorffii*, MYB43 may be more important in the MYB family, functioning in the regulation of secondary cell wall biosynthesis, which is different from *Arabidopsis* where MYB58 and MYB63 played a major role.

#### Phloem specification and differentiation

Phloem identity was thought to be established later than xylem at the end of embryogenesis [43]. Fewer genes involved in phloem formation and differentiation have been identified, compared to genes related to xylem formation and differentiation. KAN1 and KAN2, belonging to GARP family, together promote both abaxial and adaxial organ identity [45, 46]. At the early step of phloem specification, two alternative complexes existed to transpond signal from KAN1/KAN2 (Fig. 6), either OPS-BRX [47] or CLE45-BAM3 [48]. APL, NAC45/86 and NEN1-4 take part in phloem differentiation in later steps. APL encodes a MYB-type transcription factor which promotes phloem differentiation as well as suppresses xylem differentiation [20]. NAC45 and NAC86, both are targeted by APL, control the formation of sieve element cells. Further down the pathway, NEN1, NEN2, NEN3, and NEN4 regulate the enucleation process of sieve element cells [49]. However, in *S. moellendorffii*, only five phloem regulated genes, BAM3, KAN1, KAN2, NEN2 and NEN4, were identified. Our results indicated that they all expressed at low levels in the different tissues (Fig. 7c; Additional file 9). So the regulatory pathway for phloem differentiation is the least conserved pathway between the lycophytes and euphyllophytes.

#### Evolutionary trend of transcription factors for vascular development

To look into conservation of transcription factors involved in vascular development, we compared and analyzed them among four representative species, including seed and non-seed vascular plants, *Arabidopsis* and *S. moellendorffii,* and nonvascular land and aquatic plants, *P. patens* [50] and *C. reinhardtii* [51]. For each of the seven transcription factor families, a clear trend emerged that increased number of transcription factors are associated with the occurrence of the vascular system with increased complexity (Table 2). It is very likely that the transcription factors that were conserved between *Arabidopsis* and *S. moellendorffii*, but absent in both *P. patens* and *C. reinhardtii*, represent the critical elements in the evolving vascular species, and may play important roles in development of the vascular tissue. Many of them displayed distinct expression pattern from our analysis of the *S. moellendorffii* transcriptome. Such comparison of critical genes involved in vascular development offered important insight into the evolution of vascular plants, and the details of molecular mechanisms for regulation.

**Table 2:** Transcription factor families involved in vascular development in *Arabidopsis*, *S. moellendorffii, P. patens* and *C. reinhardtii*

TF family	Arabidopsis	Selaginella	P. Patents	C. reinhardtii
ANAC007	●	●	●	
ANAC010	●		●	
ANAC012	●	●		
ANAC030	●			
ANAC037	●	●		
ANAC043	●	●		
ANAC066	●	●	●	
ANAC076	●	●		
ANAC083	●	●		
ANAC101	●		●	
ANAC104	●			
ANAC105	●		●	
AFB1	●		●	
AFB2	●	●		
AFB3	●	●	●	
AFB4	●			
AFB5	●			
IAA12	●	●		
IAA20	●			
IAA28	●	●		
IAA30	●			
IAA31	●			
IAA8	●	●		
IRX1	●	●		
IRX12	●	●		
IRX14	●	●		
IRX3	●	●		
IRX9	●			
MYB20	●	●	●	
MYB42	●			
MYB43	●	●	●	●
MYB46	●	●		
MYB61	●	●		●
MYB85	●	●		
PIN1	●			
PIN3	●	●		
PIN4	●	●	●	
PIN7	●	●	●	
KAN1	●	●		
KAN2	●	●		
KAN3	●			
Total	41	26	11	2

#### Lignin biosynthesis pathway in S. moellendorffii

Lignin biosynthesis is an important mechanism in secondary cell wall growth for plant adapting terrestrial environments. There were 10 gene families involved in the lignin biosynthesis pathway in plants [52]. Previously, members of all the 10 gene families had been identified in *S. moellendorffii* [3]. In the current study, six novel lignin biosynthesis genes were found in *S. moellendorffii* (Additional file 10), and were expressed in both root, stem, and leaf tissues. The expression of Smoe_00035757, Smoe_00037872, and Smoe_00054357 was the highest in stem, and comparable in root and leaf tissues.

In addition, the PRX52 gene encoding a homologue to peroxidase, was also involved in lignin formation. It was showed that PRX52 deletion mutations had reduced synthesis of lignin and formed abnormal interfascicular fibers [53]. In *S. moellendorffii*, two genes, *235372* and Smoe_00010335 (novel coding gene), homologous to PRX52 were identified, but only Smoe_00010335 was expressed in root, stem and leaf at low levels with average FPKM value 1.63. Further, the IRX12/LAC4 gene encoding an oxidative enzyme, was found to control the lignin accumulation in secondary cell walls. IRX12/LAC4 mutants had a phenotype of ill formed xylem [54]. In *S. moellendorffii*, two genes (*165365* and *78002) *homologous to IRX12/LAC4, were found to have low expression with average FPKM values of 2.42 and 0.85. Although lignin biosynthesis in *S. moellendorffii* was reported to have difference to that in angiosperms [55], our data indicated a largely conserved synthesis pathway with high redundancy in *S. moellendorffii*.

## Conclusions

We performed deep RNA-sequencing analysis on three *S. moellendorffii* tissues. We identified 7930 high-confidence novel coding genes, and for the first time reported 4422 lncRNAs in a lycophyte. Further, we refined 2461 (11.0%) existing gene models, and identified 11,030 alternative splicing (AS) events (for 5957 coding genes) that have never been reported. Compared to higher flowering vascular plants, *S. moellendorffii* displayed a less complexity in both gene structure, alternative splicing, and regulatory elements of vascular development. The study offered important insight into the evolution of vascular plants, and the critical details in regulation of vascular development in the lycophytes.

## Methods

### Materials

Samples of *S. moellendorffii* were collected on the mountain in Tangdiyang Village, Huangtan Town, Wencheng County, Wenzhou City, Zhejiang Province, P. R. China (N27°43′39.03″, E119°59′44.34″). Roots, stems and leaves were collected respectively and frozen immediately by liquid nitrogen.

### RNA isolation, library construction and sequencing

Total RNA from the root, stem and leaf samples of *S. moellendorffii* was extracted using TaKaRa RNAiso Plus kit following the manufacturer’s instructions. The RIN number was checked to determine RNA integrity by an Agilent Bioanalyzer 2100 (Agilent technologies, Santa Clara, CA, US). Qualified total RNA was further purified using RNeasy micro kit (QIAGEN, GmBH, Germany) and RNase-Free DNase Set (QIAGEN, GmBH, Germany). rRNA was removed from the purified RNA using Ribo-Zero rRNA Removal Kit. Strand-specific RNA-seq libraries were constructed with TruSeq Stranded mRNA LT Sample Prep Kit (Illumina, USA), in which the second strand cDNA was synthesized by substituting dTTP with dUTP. Libraries were applied to Illumina Hiseq2500 platform, with paired-end sequencing length of 125 bp.

### Reads mapping, gene modeling and expression estimation

Sequencing reads were filtered according to quality score using sickle (https://github.com/najoshi/sickle), and the quality threshold was set to 20. Reads short than 50 bp or with ambiguous nucleotides were also removed after quality checking. The genome (v1.91) and gene models of *S. moellendorffii* were downloaded from JGI (https://phytozome.jgi.doe.gov/pz/portal.html#). Clean reads from root, stem and leaf were mapped onto the genome using TopHat (version 2.1.0) with default parameters, except the --library-type set as “fr-firststrand”. Cufflinks (version 2.2.1) was used to reconstruct gene models using alignment results from TopHat and the existing gene models as a guide. Gene models from root, stem and leaf were integrated into a final model using cuffmerge from Cufflinks. Existing gene models were compared with the new gene models using cuffcompare (from Cufflinks) with default parameter.

Using cuffdiff from Cufflinks, gene expression levels were estimated by computing the FPKM value (Fragment per Kilobase per Million mapped reads) [26] for transcripts. Tissue-specific genes were obtained when they were expressed in one tissue (FPKM > 0.1) and absent in both the other two (FPKM ≤ 0.1).

### Identification of novel coding genes

The following three programs were employed to estimate the coding potential of candidate transcripts. (1) The sequences of candidate transcripts and 22,285 known genes in *S. moellendorffii* were subjected to analysis by CPC tool (http://cpc.cbi.pku.edu.cn/) [27], using default settings. Coding Potential Calculator (CPC) is widely used in identifying noncoding RNAs. Statistic models of CPC were constructed according to the biological features of RNA including ORF length, ORF integrity and alignment hits with public protein database. CPC identified 21,874 of 22,285 known genes as coding potential with the minimum score of 0.86. So we selected the transcripts with scores more than 1.0 as coding potential transcripts. (2) The coding potential transcripts were subjected to TransDecoder (https://transdecoder.github.io/, version 3.0.0) to predict ORF, and only transcripts containing more than 100 amino acids were retained. (3) The public protein database Swiss-Prot were searched using blast with e-value setting as 1e-5. The strand specific RNA-sequencing can indicate the transcriptional orientation, so only the forward alignment hits were retained. (4) We also predicted the coding potential by PfamScan, and which searched the protein families collected in Pfam database based on hidden Markov models (HMMs) [56]. Taken together, transcripts with a CPC score larger than 1.0, more than 100 amino acids in length, and having hits in Swiss-Prot or Pfam were designated as high-confidence coding genes.

### Identification of noncoding RNA (ncRNA) and long noncoding RNA (lncRNA)

RNA was considered as noncoding RNA (ncRNA) from *S. moellendorffii*, when one was found to be located outside existing gene model, and had no coding potential defined using the CPC tool (http://cpc.cbi.pku.edu.cn/) [27].

A long noncoding RNA was identified using strengthened criteria: 1) ncRNA no match found in Swiss-Prot and Pfam; 2) ncRNA longer than 200 bp; 3) ncRNA with expressional level FPKM ≥0.5 for single exon, or with expressional level FPKM ≥0.1 for multiple exons.

To determine the average length, exon number, JS score, GC content, and expression level*,* the sequences of intergenic regions, and mRNA for *S. moellendorffii* were obtained from JGI database (http://jgi.doe.gov/). For coding genes, mRNAs with either average FPKM ≥0.1 for multi-exon or average FPKM ≥0.5 for single-exon were used for analysis. For control, 10,000 randomly selected intergenic sequences were used. The tissue-specific score (JS score) [41] was calculated for each transcript using the csSpecificity() function in the R’s CummeRbund package [57].

### Function annotation of novel coding genes and GO enrichment

Blast program was used to search homologies in nr, Swiss-Prot, KOG database when annotated novel coding genes, and the e-value was set as 1e-5. KEGG pathway annotation was performed by subjecting the query sequences to KAAS (http://www.genome.jp/tools/kaas/) on line. And we used PfamScan program developed by Pfam database to predict protein domains. Gene Ontology (GO) annotations were grabbed from nr alignment results and GO consortium. BinGO was employed to perform GO enrichment [58].

### RT-PCR experimental validation of novel transcripts

Total RNA was isolated from root, stem and leaf samples respectively using E.Z.N.A. Plant RNA Kit (Omega Bio-tek, Georgia, USA) and was reverse transcribed into cDNA following the manufacturer’s instructions of PrimeScript RT reagent Kit with gDNA Eraser (TaKaRa, Dalian, China). PCR reactions were performed at ABI StepOnePlus (Applied Biosystems, Foster City, USA) with an annealing temperature of 55 °C. The designed primer pairs were included in Additional file 3.

## Additional files

Additional file 1: Novel coding genes. (XLSX 1368 kb)
Additional file 2: Novel noncoding RNAs. (XLSX 634 kb)
Additional file 3: RT-PCR experimental validation of novel transcripts. (DOCX 14 kb)
Additional file 4: Modified gene models. (XLSX 460 kb)
Additional file 5: Natural antisense transcripts. (XLSX 160 kb)
Additional file 6: Alternative splicing events of known coding genes and novel coding genes. (XLSX 902 kb)
Additional file 7: Tissue-specific expression of coding genes in *S. moellendorffii.* (XLSX 131 kb)
Additional file 8: Tissue-specific expression of lncRNAs in *S. moellendorffii.* (XLSX 41 kb)
Additional file 9: Vascular development genes and their expression levels in *S. moellendorffii.* (XLSX 11 kb)
Additional file 10: The gene families of lignin biosynthesis pathways in *Arabidopsis* and in *S. moellendorffii.* (XLSX 23 kb)

## Abbreviations

Alt3′: Alternative 3′ splice site
Alt5′: Alternative 5′ splice site
Anti-lncRNA: lncRNA located on the anti-sense strand of coding genes
AS: Alternative splicing
ES: Exon skipping
FPKM: Fragment per kilobase per million mapped reads
GO: Gene ontology
IR: Intron retention
lincRNA: lncRNA in intergenic regions
lncRNA: Long noncoding RNA
MEE: Mutually exclusive exons
NAT: Natural antisense transcript
ncRNA: Noncoding RNA
