# Supplementary material for: Global transcriptome analysis reveals extensive gene remodeling, alternative splicing and differential transcription profiles in non-seed vascular plant Selaginella moellendorffii
Source: BMC Genomics. 2017 Jan 25;18(Suppl 1):1042. doi: 10.1186/s12864-016-3266-1 (PMC5310277; doi:10.1186/s12864-016-3266-1)
Supplement: Additional file 3: — RT-PCR experimental validation of novel transcripts. (DOCX 14 kb) [file 12864_2016_3266_MOESM3_ESM.docx]

| Gene ID | Forward primer | Reverse primer | PCR product | Annotation |
| --- | --- | --- | --- | --- |
| Smoe_00002040 | TTAGAAGTGCCGACAACA | CATCACCACAACTATACGC | Yes | novel coding gene |
| Smoe_00006028 | AAATGGGCGTATGGAGAT | CAAAGCGTGGTGGAACAA | No | novel coding gene |
| Smoe_00014813 | TGTGCTGATGACCCAGAC | GTAGTACCCATACAAGCTAATT | Yes | novel coding gene |
| Smoe_00015213 | TACCCGTCATGTACTTTGC | GTTCGTTTATTCAGCGATT | No | novel coding gene |
| Smoe_00015326 | GTTGGCAATGCTTATGTG | CAGAAGTAGACACGGGTAGA | Yes | lncRNA |
| Smoe_00017617 | TGTTGCTGCCGACGATGA | CACTTGGATGACGGTTTGG | Yes | lncRNA |
| Smoe_00017948 | TCATGGCTGGGATGTTTG | CGAGGATGGTGGTGGACT | Yes | novel coding gene |
| Smoe_00024240 | CATCATCCATCCCAGAAA | GAGTAGCTCGCCACCATT | Yes | novel coding gene |
| Smoe_00030006 | GCAAATGCCCAGTTTCAG | GGAGCCAATCCGATGTTA | Yes | lncRNA |
| Smoe_00032648 | TGAGATGGAGCCTGAGTG | GCAGGACCATAAGCACTAA | Yes | novel coding gene |
| Smoe_00039112 | TGTTTATGAGGGTGGAGA | CAGGTAGGAGACTGGAGC | Yes | lncRNA |
| Smoe_00041910 | GGTGGCTTACTAGGAGGCG | CTGTGGAGCGAGGATTGG | Yes | novel coding gene |
| Smoe_00048044 | GGAGGAGCAACCAATAAA | TCCGGGAGAATCATGTAC | Yes | novel coding gene |
| Smoe_00049493 | TTTGGATCGCAGTGCTTACC | GGCAGCGGGATTTGTCTT | Yes | novel coding gene |
| Smoe_00054042 | ACGGGTACAAACATTGAG | ATGCCATTCGTGTTACTT | Yes | lncRNA |
| Smoe_00031191 | GTGTTGCGGCTTTGCTTA | CCCACCAGGTTGGAGATT | No | ncRNA |
| Smoe_00003935 | AGTCGCTTGCCCACTTGC | CGCTCCTTTTCCCTTTGA | Yes | novel coding gene |
| Smoe_00027071 | AAGACTCGCCATCGGACAT | TCGTTTCATACGCCTTCG | Yes | novel coding gene |
